# An integrative review of home care recommendations for women after caesarean section

**DOI:** 10.1002/nop2.2145

**Published:** 2024-03-26

**Authors:** Mwajuma Bakari Mdoe, Lilian Teddy Mselle, Stephen Mathew Kibusi

**Affiliations:** ^1^ Department of Clinical Nursing, School of Nursing and Public Health University of Dodoma Dodoma Tanzania; ^2^ Department of Clinical Nursing, School of Nursing Muhimbili University of Health and Allied Sciences Dar es Salaam Tanzania; ^3^ Department of Public Health, School of Nursing and Public Health University of Dodoma Dodoma Tanzania

**Keywords:** discharge information, home care after caesarean delivery, post‐caesarean delivery, recommended home care, surgical site infection

## Abstract

**Aim:**

To describe home‐based care and practices recommended for mothers after caesarean section (CS), existing in the literature.

**Design:**

Systematic review.

**Methods:**

This integrative literature review was conducted by using Google Scholar, PubMed and Hinari databases from its inception to June 2021. The search included research articles, protocols and guidelines that describe home care practice after CS and assessed for their quality. Synthesis of recommendations from the included literature was classified based on the type of study design, and the review was guided by methodology registered in Prospero (ID CRD42021276905).

**Results:**

A total of 681 literatures were found, and 12 met the criteria. Recommended home care components were divided into four major categories; wound care practice (wound cleaning, drying and bandage change); nutrition (high‐protein diet, vitamin C, fibre and balanced diet); exercise (pelvic floor muscle, walking and breathing exercise) and hygiene (bathing and wearing clean clothes). There was limited literature addressing the components of perineal care and home environment. Varying recommendations were found regarding wound cleaning and dressing in the studies done in low‐ and high‐income countries.

## 1 INTRODUCTION

Globally, the rate of caesarean deliveries has increased from 12% in 2000 to 21% in 2015 (Boerma et al., 2018). Despite the fact that caesarean section (CS) is a lifesaving intervention, it is associated with significant risks and complications including, surgical site infection (SSI) (Field & Haloob, 2016; Nyamtema et al., 2016; WHO, 2019). The SSI is the most common complication of CS and contributes to maternal morbidity and mortality (Shahane et al., 2012). For example, 20% of mothers globally who deliver by CS develop post‐operative wound infections (World Health Organization (WHO), 2018). In Tanzania, between 11% and 48% of mothers develop SSI after a caesarean birth (De Nardo et al., 2016;Mpogoro et al., 2014; Nguhuni et al., 2017).

The SSI post‐CS occurs due to various factors including health conditions in pregnancy and labour (De Nardo et al., 2016; Mpogoro et al., 2014; Triantafyllopoulos et al., 2015), use of unclean equipment during obstetric procedures, mother's care and practices at home (Mpogoro et al., 2014; Nguhuni et al., 2017), inadequate preparation and care after CS, and lack of use of standard care guidelines including guideline of care after hospital discharge (Campa, 2020; Nwankwo et al., 2012; Triantafyllopoulos et al., 2015).

Infection prevention and control (IPC) is a core responsibility of midwives and nurses, essential to the prevention of maternal and newborn sepsis, especially in the current time of antimicrobial resistance (Sickder et al., 2017; World Health Organization (WHO), 2019). Evidence shows that nurses and midwives have varying knowledge, low attitude and practice towards the prevention of SSI (Mohsen et al., 2020; Oluwakemi et al., 2017; Sickder et al., 2017). Nurses and midwives need to be knowledgeable about the recovery requirements of post‐operative patients in order to assist them in the process. Frequency updating of their knowledge with guidelines, adequate staffing and follow‐ups in the implementation of guidelines will help in SSI prevention practices.

Between 2017 and 2019 different organizations provided recommendations for preventing SSI of all types of surgical procedures and caesarean deliveries in the healthcare environment (Berriós‐Torres et al., 2017; Macones et al., 2019; WHO, 2018a). However, those organizations did not provide home care recommendations for mothers after CS for the prevention of SSI. For example, authors of ERAS Society guidelines acknowledged the limitation of evidence on discharge counselling for mothers after CS (Macones et al., 2019).

Consequently, mothers in many settings receive little information about home care practices after CS (Campa, 2020), despite the fact that many mothers are discharged early from postnatal wards to create space for new delivery mothers (Atuhaire, 2021). This evidence emphasizes the need to guide the care of mothers at home after CS to prevent SSI and promote full recovery; especially in Tanzania (Mdoe et al., 2023) where home visit by healthcare personnel to assess the progress of recovery is not routinely done after hospital discharge (Campa, 2020).

There are scattered information from literature about homecare and practices to enhance recovery after CS that can be used in the systematic review as the first step in the development of the home care guide after CS in Tanzania (American College of Surgeons, 2018; Campa, 2020; Mohamed et al., 2019; University Hospital Bristol, 2019). The developed guide can be used by healthcare providers such as nurses and midwives to advise mothers about their care and practice at home following CS deliveries. Hence, the aim of this study is to conduct an integrative review of home‐based care and practices recommended for mothers after CS.

In 1854, Florence Nightingale recognized the importance of the environment in wound care and healing (Fee & Garofalo, 2010; Medeiros et al., 2015). Such an environment emphasized key infection prevention measures and healing, such as hygiene, clean water and sanitation, personal cleanliness, optimal ventilation and lighting, optimal nutrition, and chattering hope and advice (Medeiros et al., 2015). For this review, Nightingale's theoretical framework guided the review of home care recommendations, especially for (1) home‐based wound care practices to prevent SSI, (2) nutritional practices to enhance recovery, (3) hygienic practices to prevent SSI, (4) exercises and rest to enhance recovery and (5) home environmental factors to enhance recovery of post‐CS mothers.

## 2 METHODOLOGY

This integrative review was conducted to identify and synthesize contents and components of post‐CS home care recommended practices existing in the literature. The integrative review was used because it offers a comprehensive understanding of home care after CS as it includes empirical and theoretical literature and research that has varied designs (Oermann & Knafl, 2021). This design is useful especially when the topic under study is not extensively studied.

The six stages of the review process put forward by Souza et al. (2010) were used, and the review was guided by methodology registered in Prospero with the ID ‘CRD42021276905’. These include (1) preparing the guiding questions, (2) searching or sampling the literature, (3) data collection, (4) critical analysis of the studies included, (5) discussion of results and (6) presentation of the integrative review.

### 2.1 Guiding questions

The guiding questions were as follows:

1. What are home‐based wound care practices recommended for preventing SSI among post‐CS mothers?
2. What are nutritional recommendations for enhancing recovery among post‐CS mothers?
3. What are the home‐based hygienic practices recommended for preventing SSI among post‐CS mothers?
4. What are the home‐based exercises recommended to enhance recovery among post‐CS mothers?
5. What are the home environmental recommendations to enhance recovery among post‐CS mothers?

### 2.2 Search words

The guiding questions above were used to search literature according to MeSH terms, as seen in Table 1.

**TABLE 1:** Guiding questions and search words.

S/N	Guiding questions	Search words
1.	What are home‐based wound care practices recommended for preventing SSI among post‐CS mothers?	(‘surgical site infection’ OR ‘SSI’ OR ‘wound sepsis’ OR ‘wound infection’ OR ‘wound colonization’ OR ‘wound contamination’) AND (‘wound care’ OR ‘wound care instructions’ OR ‘wound hygiene’ OR ‘surgical site care’ OR ‘bandage change’) AND (‘post caesarean section mothers’ OR ‘caesarean mothers ˮ OR ‘post‐operative women’ OR ‘post caesarean birth’ OR ‘caesarean birth’)
2.	What are nutritional recommendations for enhancing recovery among post‐CS mothers?	(‘caesarean section recovery’ OR ‘attaining health’ OR ‘post‐partum recovery’ OR ‘caesarean section wound healing’) AND (‘nutritional recommendation’ OR ‘nutritional guide’ OR ‘diet’ OR ‘nutrition’ OR ‘home care interventions’) AND (‘post caesarean section mothers’ OR ‘caesarean mothers’ OR ‘post‐operative mothers’ OR ‘post caesarean birth’ OR ‘caesarean birth’)
3.	What are the home‐based hygienic practices recommended for preventing SSI among post‐CS mothers?	(‘surgical site infection’ OR ‘SSI’ OR ‘wound sepsis’ OR ‘wound infection’ OR ‘wound colonization’ OR ‘wound contamination’) AND (‘hand hygiene’ OR ‘bathing’ OR ‘general hygiene’ OR ‘perineal care’ OR ‘hygienic recommendation’) AND (‘post caesarean section mothers’ OR ‘post‐operative mothers’ OR ‘caesarean mothers’ OR ‘post caesarean birth’ OR ‘caesarean birth’)
4.	What are the home‐based exercises recommended to enhance recovery among post‐CS mothers?	(‘caesarean section recovery’ OR ‘attaining health’ OR ‘recovery’ OR ‘postpartum recovery’ OR ‘caesarean wound healing’) AND (‘recommended exercise’ OR ‘walking’ OR ‘exercise’ OR ‘Kegels exercise’ OR ‘abdominal exercise’) AND (‘post caesarean section mothers’ OR ‘caesarean mothers’ OR ‘post‐operative mothers’ OR ‘post caesarean birth’ OR ‘caesarean birth’)
5.	What are the home environmental recommendations to enhance recovery among post‐CS mothers?	(‘caesarean section recovery’ OR ‘attaining health’ OR ‘recovery’ OR ‘postpartum recovery’ OR ‘caesarean wound healing’) AND (‘home environment’ OR ‘environmental ventilation’ OR ‘environmental light’ OR ‘environmental health’ OR ‘environmental cleanness’) AND (‘post caesarean section mothers’ OR ‘caesarean mothers’ OR ‘post‐operative mothers’ OR ‘post caesarean birth’ OR ‘caesarean birth’)

### 2.3 Searching or sampling of literatures

From the time the databases were created until June 2021, a variety of databases, including PubMed, HINARI and Google Scholar, were used to conduct a systematic literature search.

This search included empirical literature (research articles) and secondary literature (protocols and guidelines) that provide components, instructions or procedures for post‐CS home care. Studies searched included interventional, observational and qualitative studies. The protocol and guidelines from recognized hospitals were also included. Literature that was not in the English language were excluded.

### 2.4 Data collection

The validated tool by Ursi (2005) was used to gather relevant information from selected literature that helped to minimize the risk of errors, ensured precision and saved a record after documentation (Souza et al., 2010). The literature was included if it reports population of interest (post‐CS mothers) under which the guideline, description of the type of the literature, home care intervention or recommendation and outcome of interest (SSI). Two authors independently screened the titles and abstract based on the set criteria and agreed on the selected papers after critical analysis and discussion among the authors.

### 2.5 Critical analysis

Evaluation of quality of the literature included was observed (Hawker et al., 2002; Whittemore & Knafl, 2005). The evaluation of quality of data used a two‐point scale, that is, data relevance and methodological rigour (Hawker et al., 2002; Whittemore & Knafl, 2005). However, the guidelines and case reports were not assessed for quality. The relevance of data was based on the appropriateness of the research aim and the methodological rigour evaluated clarity of description of the method, use of sampling, ethical issues and analysis of data (Sullivan & Asselin, 2013). Studies were rated one (low) if it had lower rigour or relevance to the chosen questions; and two (high) if it had acceptable rigour and relevance were rated high. No studies were excluded because of having low or higher rank (Sullivan & Asselin, 2013).

Thematic analysis was used to analyse data (Dhollande et al., 2021; Oermann & Knafl, 2021) from different types of studies, guidelines and protocols; and interpreted to understand the home care guidelines and recommendations and their effect on SSI. The recommendations were classified based on the design type suggested by Ursi (2005) that are Level 1: evidence resulting from meta‐analysis of multiple randomized controlled clinical trials; Level 2: evidence from individual studies with experimental design; Level 3: evidence from quasi‐experimental studies; Level 4: evidence of descriptive (non‐experimental) studies or with a qualitative approach; Level 5: evidence from case reports or from experience and Level 6: evidence based on opinions of specialists (Souza et al., 2010).

### 2.6 Research ethics committee approval

This study received research ethics committee approval from the Muhimbili University of Health and Allied Science Institutional Review Board (no DA.282/298/01.C/).

## 3 RESULTS

### 3.1 Search outcome

**FIGURE 1:**
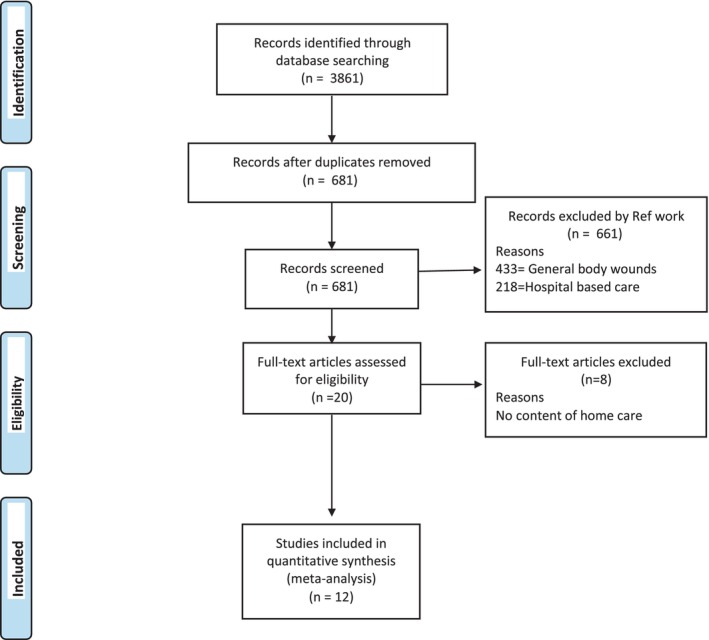
PRISMA flow diagram of the literature search, identification, screening and selection. *Source*: PRISMA flow chart.

The study used three databases of PubMed, HINARI and Google Scholar that yielded 3861 items for the first search words. Twelve literature published between 2011 and 2020 met the inclusion criteria and were obtained for a review (Figure 1: PRISMA flow diagram of the literature search, identification, screening and selection). Among the 12 included literatures, there were eight experimental studies, two hospital guidelines, one qualitative study and one case report, as shown in Table 2. Summary of the results of the included study is shown in Table 3.

**TABLE 2:** Summary of the home care recommendation and the level of evidence of the included studies.

Author (s)/year	Type of literature	Study design	Wound care	Medication	Danger sign	Nutrition	Hygiene	Exercise and rest	Level of evidence	Quality of the included literature
Ibrahim et al. (2011)	Article	RCT	√						1	2
Hussien et al. (2021)	Article	Quasi‐Experimental study	√	√	√	√		√	3	1
Campa (2020)	Thesis	Qualitative study	√	√	√			√	4	2
Aarthi (2012)	Thesis	Experimental study				√		√	2	1
Darmawati et al. (2019)	Article	Experimental study				√			2	1
University Bristol Hospital (2019)	Guideline	–	√		√	√			6	N/A
Ryan (2016)	Guideline	–	√		√		√		6	N/A
Purba et al. (2020)	Article	Quasi‐experimental study				√			3	2
Hicks‐roof (2018)	Case report	–				√			5	N/A
Robb et al. (2020)	Article	RCT							1	2
Thakur (2020)	Thesis	Quasi‐Experimental study						√	3	2
Vijaya Rani et al. (2016)	Article	Experimental study						√	2	2

**TABLE 3:** Summary of the methodology and results of each included study.

Author's name, year	Type of literature	Study design	Participants, sample size	Intervention	Follow up	Results
Ibrahim et al. (2011)	Article	RCT	200	Prophylactic antibiotics, Cephalosporin versus Amoxicillin to pregnant women; and wound care education	10 days post‐operative	Amoxicillin is as effective as Cephalosporin in the prevention of SSI. women who received health education about wound care are less likely to be exposed to wound infection than those who received only routine hospital care
Hussien et al. (2021)	Article	Quasi‐Experimental study	250 women undergoing CS	Women were divided into two identical groups; the study group received the post‐operative Enhanced recovery after Surgery (ERS) in addition to routine hospital care and the control group received the routine hospital care	14 days	Post‐CS mother who were in intervention study had an effect on relieving the women's post‐operative problems especially pain than those who received the routine hospitals nursing care only
Campa (2020)	Thesis	Qualitative study	10 clinicians and 10 post‐CS mothers	NA	NA	Home care education component: Bandage change, bathing, changing clothes, restricted physical effort, and when to return to a health facility for follow‐up
Aarthi (2012)	Thesis	Experimental study	60 mothers admitted for elective CS	Effectiveness of pre‐operative teaching on post‐operative outcome among women who undergo elective CS	From admission to post‐operative day 7	The pre‐operative teaching was effective in reduction of complication of caesarean section and in obtaining adequate health status of the mothers. Teaching on diet, postnatal exercise, breast feeding, hygiene, newborn care and complications.
Darmawati et al. (2019)	Article	Experimental study	30 post‐CS mothers	Effectiveness of high‐protein nutrients intake for post‐CS wound healing process	7th day	High protein to post‐CS patients lead to high proliferative wound healing than the control group
University Bristol Hospital (2019)	Guideline	–	N/A	Tips for recovery after a caesarean	N/A	Getting help from friends and family, activity and rest, wound care, driving, avoiding constipation, family planning and recovery time
Ryan (2016)	Guideline	–	N/A	Creation of discharge instructions for the specific population of obese post caesarean women going home with negative pressure wound therapy was needed in order to properly educate these women and to further prevent infection complication	N/A	Guideline with the following component was formed; physical activities, hygiene, elimination and bowel movement, nutrition, fever and pain detection and control, incision care, vaginal care, sexual intercourse, breast care, mental health care
Purba et al. (2020)	Article	Quasi‐experimental study	34 post‐CS mothers	Effect of Giving Gabus Fish on the Healing Process of Post‐operative Section Caesarean	14th day	Gabus fish consumption can accelerate healing post‐operative wounds of caesarean section around 84%
Hicks‐roof (2018)	Case report	N/A	1 post‐CS mother	Arginine‐enriched oral nutrition supplement twice daily as a therapeutic intervention to aid in wound healing and recovery	14 days	Consuming an arginine‐enriched oral nutrition supplement optimize healing following a CS
Robb et al. (2020)	Article	RCT	173 post‐CS mothers	Availability of WASH conditions in the post‐partum ward of a district hospital over 2 months, the WASH conditions at the women's homes, and the association between WASH conditions and suspected surgical site infection	2 months	Women exposed to a day or more without running water in the hospital were 2.6 times more likely to develop a suspected SSI
Thakur (2020)	Thesis	Quasi‐Experimental study	500 post‐CS mothers	Planned structured exercise session was initiated for the experimental group on the day of lower segment caesarean section and was followed twice a day for the first 5 post‐caesarean days	5th day post‐operative	Structured post‐CS exercise sessions are effective in reducing the potential post‐operative problems associated with immobility in post‐caesarean period
Vijaya Rani et al. (2016)	Article	Experimental study	50 post‐CS mothers	Planned teaching programme on practice of postnatal exercises among mothers who have undergone Lower segment CS	14 days	Control group had inadequate practice score compared to experimental group, the difference was statistically significant

Of 12 literatures included, five described wound care practices at home for mothers after they had CS deliveries (Campa, 2020; Hussien et al., 2021; Ibrahim et al., 2011; Ryan, 2016; University Hospital Bristol, 2019). They provided recommendations for wound cleaning and management.

#### 3.1.1 Wound cleaning and dressing

A range of wound care practices were recommended following a CS. Mothers were advised to clean the wound with soap and water during bathing and then dry the wound with a clean towel or piece of cloth (Hussien et al., 2021; Ryan, 2016; University Hospital Bristol, 2019). If a dressing is present, the dressing should be kept dry and the woman should also avoid soaking the wound or immersion in water during swimming or bathing (Ibrahim et al., 2011). Another study clearly recommended the use of sponge baths to prevent the wound from being soiled and to promote wound healing (Campa, 2020). One study also recommended use of antiseptic solution to clean the wound before changing the dressing (Ibrahim et al., 2011). While one study has recommended the dressing of the wound to be performed at home (Ibrahim et al., 2011), another study recommended that wound dressing to be done in the health facility after every 2–3 days rather than at home (Campa, 2020). Other studies recommended dressing of the wound only if draining or irritated, otherwise the incision should be left without dressing (Ryan, 2016). Studies also reported the need for the women to be informed about signs of wound infection including; increased redness, drainage, swelling or separation of skin (Campa, 2020; Hussien et al., 2021; Ryan, 2016).

#### 3.1.2 Adherence to post‐operative medication

Two of the 12 literatures recommended adherence to medication such as pain relief and prophylactic antibiotics prescribed to mothers after CS (Campa, 2020; Hussien et al., 2021). They were also recommended to compress/hold the wound site when coughing and standing to relieve pain (Ibrahim et al., 2011).

#### 3.1.3 Restriction of activity

Three studies recommended that mothers after CS should restrict activities (Campa, 2020; Hussien et al., 2021; Ibrahim et al., 2011). They should avoid lifting heavy items 2–3 weeks after CS and swimming (Campa, 2020; Hussien et al., 2021; Ibrahim et al., 2011).

#### 3.1.4 Nutrition to enhance mothers' recovery after CS

Nutritional recommendations were given by six of twelve studies reviewed (Aarthi, 2012; Darmawati et al., 2019; Hicks‐roof, 2018; Ibrahim et al., 2011; Purba et al., 2020). To promote wound healing, mothers should be advised to eat high‐protein diet such as eggs, albumin protein such as Gabus fish and arginine supplements (Aarthi, 2012; Darmawati et al., 2019; Hicks‐roof, 2018; Ibrahim et al., 2011; Purba et al., 2020). Mothers should take a well‐balanced diet constituting 2600 kcal, protein 65 g, iron 30 g, calcium 1500 mg, zinc19 mg, vitamin A 8000 IU, vitamin D 400 IU, thiamine 1.5 mg, riboflavin 1.5 mg, nicotinic acid 17 mg, ascorbic acid 70 mg, folic acid 400 μg, vitamin B_12_ −2 μg and fibre (Aarthi, 2012). Further, to improve body immunity and promote wound healing mothers post‐CS should increase intake of vitamin C and iron supplementation (Ibrahim et al., 2011).

#### 3.1.5 Hygienic practices for preventing SSI

Six studies have recommended hygienic practices for post‐CS mothers including showering, washing hair and taking tub baths (Campa, 2020; De Nardo et al., 2016; Hussien et al., 2021; Ibrahim et al., 2011; Robb et al., 2020; Ryan, 2016). A study done by Robb et al., 2020, that assessed the association between WASH (safe water, sanitation and hygiene) conditions and suspected surgical site infection (SSI) at home found that mothers with improved sanitation and water supply source were less likely to develop SSI compared with the mothers who had unimproved sanitation and water supply. The study recommended improved sanitation and water supply as a means of preventing SSI (Robb et al., 2020). Post‐CS mothers were also advised to wear clean clothes after bath, avoid becoming in contact with dirty (Campa, 2020), douching or use tampons for 3 weeks after CS to minimize risk of infection when caring for the perineum (Ryan, 2016).

#### 3.1.6 Conducting exercises to enhance recovery of post‐CS mothers

Four studies recommended post‐CS mothers to conduct a variety of exercise to enhance healing (Hussien et al., 2021; Vijaya Rani et al., 2016) (Aarthi, 2012; Thakur, 2020). Recommended exercises include basic foot and leg exercises, breathing and coughing exercises, abdominal breathing exercises, hip hitching and pelvic tilt exercises, and knee rolling exercise. Additional guidance to perform Kegel exercises to strengthen the pelvic floor are also shared. Other exercises to enhance recovery included walking, use of good posture and light stretching (Hussien et al., 2021).

## 4 DISCUSSION

The current literature review revealed that there are no established guidelines for post‐CS home‐based care. However, there are documented practices that vary across countries; for example, in developed countries, home care practices of wound cleaning by using soap and water were recommended while the same was strongly prohibited in literature from developing countries. The current review employed an integrative review of literature, guided by Nightingale's environmental theory to arrive at the key questions and search words in credible sources including PubMed, Google scholar and Hinari. The integrative analysis employed six stages of Souza et al. (2010) to arrive at the most reported home care practices for the prevention of SSI. Overall, there is limited literature about this topic in terms of number, quality and depth of analysis and methodologies employed.

To enrich the evidence, we reviewed other available possible sources including hospital guidelines and protocols on home care after CS. Our integrative review included all available possible sources including hospital guidelines, protocol and case reports to support the development of recommendations for home care practice in preventing SSI to post‐CS mothers. Among the included literatures, there is no guideline or hospital protocol from a developing country that addresses home care after hospital discharge after CS mothers. Hospital protocols for home care practices in developed countries were available (University Hospital Bristol, 2019) but evidence and guidance are lacking for low‐resource settings, such as Tanzania.

### 4.1 Wound care

The literature is mixed in terms of recommendations for wound care after CS. In some settings, washing the wound with soap and water during bathing was recommended for after CS (Hussien et al., 2021; Ryan, 2016). However, wound care recommendations in Egypt and Haiti are different than in Bristol, UK (Campa, 2020; Hawker et al., 2002; University Hospital Bristol, 2019). In low‐resource settings, SSI prevention involves keeping the wound dry and covered. In high‐resource settings such as the UK, post‐CS mothers may shower normally 24–48 hours after CS (University Hospital Bristol, 2019). In Tanzania, post‐CS mothers are advised to avoid water on their wounds while bathing for a full 7 days and typically the skin layer has mostly healed by that point. The woman will then be allowed to resume bathing, typically a sponge bath while covering the wound with a clean cloth (on top of the bandage to ensure no water contacts the wound) is recommended. Bathing and wound care guidelines in low‐ and high‐resource settings are quite different, likely due to the skin closure approaches and bandages available in developed countries. Access to clean water for bathing is also a major challenge in developing countries, thus restricting bathing until the skin layer is healed is a key strategy for prevention of SSI (University Hospital Bristol, 2019). Due to an inadequate supply of clean water in Tanzania, especially in rural areas, wetting the wound with water during bathing might predispose the wound to infection. There is an importance of improving adequate supply of clean water and the environment sanitation to post‐CS mothers and the whole community that will enhance hygienic practices like hand washing, cleaning clothes and the environmental cleanness as a whole.

In Canada, it is recommended that after CS, mothers clean the wound with an antiseptic solution when changing the gauze at home if found wet (Ibrahim et al., 2011). This is supported by the British Columbia Provincial Nursing Skin and Wound Committee, the guideline for assessment and treatment of wound infection (British Columbia Provincial Nursing Skin and Wound & Committee, 2017). According to the guideline, patients and family members are educated about when and how to change the bandages at home and taught proper dressing techniques. In contrast, a study conducted in Haiti recommends that all activities involving gauze changing be performed in a hospital environment where a sterile environment is guaranteed (Campa, 2020). Similarly, in Tanzania, all activities involving bandage change or wound dressing are done in the hospital by skilled personnel and under sterile techniques. Even after recognizing the bandage is wet they are advised to go to the hospital for proper management including wound assessment and bandage change. In developing countries such as Haiti and Tanzania, it is often challenging to find a sterile environment for wound care at home and wound dressing activities are therefore performed in the hospital setting to reduce the risk of SSI. Such an approach also ensures wound assessment by a trained health worker. Access to sterile bandages and antiseptic solutions may also not be feasible in Tanzania or Haiti as families may not be able to afford the cost of such specialty items.

Post‐operative medication has also been recommended for wound care to post‐CS mothers (Mohamed et al., 2019; University Hospital Bristol, 2019). The aim is to prevent wound infection and reduction of pain (Macones et al., 2019) to enhance wound healing and ability to return to daily functional activities of post‐CS mothers including care of the baby. The practice is the same as in developing country like Tanzania where post‐CS mothers are discharged by post‐operative drugs containing antibiotics and analgesics to be used at home. Post‐CS mothers should be counselled to adhere to these medications as antibiotics will prevent SSI and analgesics will prevent post‐operative pain (Macones et al., 2019; World Health Organization (WHO), 2018) and enhance their mobility. Counselling of dosage, frequency and rationale of the prescribed medication is important in promoting adherence of the prescribed medication, as the crucial component for post‐CS home care guide.

### 4.2 Physical activities and rest

Exercise has also been described as an important home care component for post‐CS mothers (Aarthi, 2012; Hussien et al., 2021; Vijaya Rani et al., 2016; Thakur, 2020). Key exercises cited include: Kegels, walking, breathing exercises and gentle stretching. Description of exercises and duration is important to guide post‐CS mothers on how to do it. Early and progressive ambulation has been emphasized after CS section as this reduces the post‐operative risk (Vijaya Rani et al., 2016) of thromboembolism (Bonney, 2010). Additionally, exercise after CS has been found to improve mobility, reduce pain, strengthen muscle tone, promote involution of the uterus, support lochia drainage, promote gastrointestinal and urinary tract function, and enhancement of wound healing (Ain et al., 2018; Rana et al., 2019). Most mothers in Sub‐Saharan countries lack knowledge on post‐CS exercise to enhance their healing. A study conducted in Uganda at Mbarara Referral Hospital on assessing knowledge and practices of post‐CS mothers towards self‐care after delivery found only 21.3% of the study population had knowledge about post‐CS exercise (Atuhaire, 2021). Similar results were found in South Africa at a tertiary hospital where 32% of study participants had knowledge about post‐CS exercise, and among those only 8% practised post‐CS exercises (Mbombi et al., 2017). Worldwide, there are many cultures and traditions that address the post‐partum period, especially in Africa.

Despite the importance of the exercise, studies have highlighted restriction of activities as the component of home care practices to post‐CS mothers. Post‐CS mothers are recommended to avoiding lifting heavy objects for the first 2–3 weeks after CS, swimming, exposure of the wound to sun rays and heat; sitting in hot water, covering the body with tight clothes (Campa, 2020; Hussien et al., 2021; Ibrahim et al., 2011). This is supported by the enhanced recovery guideline after CS that activities that will involve abdominal muscles will lead to loosening of CS sutures (Adshead et al., 2020) and causing wound gaping that may result in bleeding, insufficient wound healing and high risk of wound infection. Instead, rest has been encouraged that will help to prevent post‐partum fatigue and mood changes, and will help to increase bonding between a mother and a baby; also will help injured muscles and recover and prevent oedema (Adshead et al., 2020). Rest also promotes relaxation to support lactation (Ebrahim & Esmat, 2018); hence, it is an important component for post‐CS home care, and it is applicable in Tanzania.

Despite the advantage of resting mentioned above, prolonged rest is a common traditional practice for post‐partum mothers (Dennis et al., 2007) including post‐CS mothers. This practice contradicts the scientific evidence and increases the risk of thromboembolism (Adshead et al., 2020; Bonney, 2010). Post‐CS mothers can be encouraged to perform light chores at home as a way of avoiding a prolonged period of resting. Depending on the cultural context, clear and careful messaging may be needed. There is a need to educate mothers who are recovering from CS about a recommended activity level as they recover and heal. Understanding local culture and tradition is vital as such messaging and guidelines are created to educate the community.

### 4.3 Nutrition

Regarding nutrition, protein and vitamin C have been recommended to post‐CS mothers in the included studies (Aarthi, 2012; Darmawati et al., 2019; Hicks‐roof, 2018; Ibrahim et al., 2011; Purba et al., 2020). A study done by Darmawati et al. to test the effectiveness of high‐protein nutrient to post‐CS wound healing process found a statistically significant differences of post‐CS wound healing process on intervention and control group, such that wound healing is better in the group with a high‐protein diet compared to a group with normal nutrients (Darmawati et al., 2019). Protein is an essential micronutrient that plays an important role for tissue maintenance and repair, it also helps wound tissue depletion by reducing the formation of fibroblasts and the development of collagen (Darmawati et al., 2019). Lack of protein will affect the wound healing process by inhibiting the fibroblastic response, the formation of new blood vessels and the synthesis of collagen (Greyligh, 2010).

Vitamin C increases body immunity and enhances wound healing (Aarthi, 2012; Ibrahim et al., 2011). The St Michael's hospital guideline highlighted plenty of fruit, vegetables, whole grains and other foods high in fibre, plenty of fluid to be taken to avoid constipation to post‐CS mothers (University Hospital Bristol, 2019). It is important to include fibre in the diet while recovering from surgery, fibre plays a major role in preventing constipation, a common complication after surgery (European Wound Management Association, 2020). The hot and cold food belief is universal and is underpinned to most diseases and illnesses in the society, especially in Indian, Malay, Korean, Sinhalese and Mexican cultures, Latin American, North American, European and African countries as well (Acharya, 2013). There is a practice of avoiding some foods during illness. Patients will definitely follow these food beliefs for the aim of attaining quick recovery. For example, the foods that are hot have been reported to be avoided. Foods like pulses (an annual leguminous crop) are particularly avoided especially after a surgery because of the belief that they cause the development of ‘pus’ at surgical sites or wounds (Acharya, 2013). In India, there are ‘Myths and facts about Caesarean section’; most mothers and mothers‐in‐law have dietary restrictions like avoiding milk, ghee, rice in post‐CS as this can impair healing (Acharya, 2013). Another study conducted in India by Acharya to post‐operative patients found that about 98% of study respondents have food restrictions after surgery. Among those, 96% mentioned a black dal, 67% mentioned meat and/or fish, 65% mentioned potatoes and 43% mentioned tomato as a restricted food (Acharya, 2013). In the same study, 80% of study participants mentioned citrus fruits, 34% mango, 10% banana as the fruits that they restricted to take during post‐operative period; great reasons being they cause pus in the wound. Only 2% of the study participants mentioned all types of food can be taken during post‐operative period (Acharya, 2013). Dietary restriction to post‐CS mothers will affect the wound healing as food nutrients like proteins and vitamins are highly needed to enhance recovery by boosting body immunity and synthesizing new cells. Dietary restriction will also lead to a nutritional disorder to post‐CS mothers of childbearing age (Darmawati et al., 2019), and hence affect breast feeding of newborn babies. Dietary recommendations highlighted in the included article are available in our settings; hence, post‐CS mothers and their families are needed to be counselled with the nutritional requirement in post‐partum period that will enhance wound healing and quick recovery.

### 4.4 Hygiene

Hygiene has also been recommended practice to prevent SSI to post‐CS mothers. This includes bathing/sponge bath, cleaning hair and clean clothes (Campa, 2020; Mohamed et al., 2019; Robb et al., 2020; Ryan, 2016). Hygienic practices help to prevent introduction of organisms to the wound and hence prevent SSI. Perineal hygiene is not among the recommended home care practice to post‐CS mothers from the included literatures but it is a very important component of home care practices with regard to lochia that occurs after both vaginal and CS births (Mahalakshmi, 2018). Perineal hygiene has been recommended in the study done in India by Mahalakshmi on effectiveness of betadine and chlorhexidine on reducing the occurrence of urinary tract infection among mothers with indwelling catheter in post‐operative caesarean. The recommendation involves daily cleaning of external genitalia and anus, to promote cleanliness and prevent infection and also to remove irritating odorous secretions on the inner surface of the labia (Mahalakshmi, 2018). It is advised to always use the front to back manner in cleaning the perineum (Mahalakshmi, 2018), to avoid contamination of the vulva with the bacteria from anus. In the study performed at a Baghdad teaching hospital to assess effectiveness of women's self‐care instructions after CS highlighted a range of key perineal care practices. These included: washing hands before and after using the bathroom, washing the perineal area with warm water and an antiseptics from front of the pubic bone to the anus, use of clean sanitary pads to protect the perineal area from contamination, changing of sanitary pads with each urination and defecation and abstaining for sexual intercourse during the first 6 weeks after CS (Kadhim, 2018). These hygienic recommendations can be applied in most settings to prevent unnecessary infections to post‐CS mothers.

### 4.5 Environmental surroundings

No studies that addressed home environmental factors to enhance recovery were found. However, there were some studies that addressed the impact of the hospital environment on general wound healing irrespective of CS status; in regard to environmental ventilation (WHO, 2018b). Our search did not uncover published studies addressing the home environment and its importance in wound healing for post‐CS mothers.

## 5 CONCLUSION

From the reviewed literature in this study, five components and content of home care and practice were found like restricting water into the wound, hospital bandage change, hygiene, nutrition, exercises and post‐CS medication adherence that can help in development of post‐CS home care guide. A range of home care practices were described but we uncovered a clear gap in both the evidence for post‐CS home care and a lack of clear evidence‐based guidelines for mothers in low‐resource settings. The home care recommendations obtained from literature differ greatly from one setting to another and at present there is no single guideline or study for low‐ and middle‐income countries that comprehensively addresses post‐operative home care after CS. This makes some recommendations difficult to apply in developing countries such as Tanzania and masks a clear evidence‐based home care guide after CS for middle‐ and low‐income countries (Lurain, 2010). The post‐CS home care guideline must also be addressed. Further studies are needed to explore perspectives of mothers and health experts on home and in developing evidenced‐based home care guidelines to prevent significant morbidity and mortality from SSI among mothers undergoing CS in low‐ and middle‐income countries.

### 5.1 Limitations of study

In this review, limited number of literature was found about home care recommendations after CS, in numbers and quality of the involved studies. Despite that, authors managed to review the literature using this naïve method (integrative) that was necessary in the comprehensive understanding, gathering and synthesis of results.

## AUTHOR CONTRIBUTIONS

All authors participated in designing the study, literature search, analysis and preparation of the manuscript. Second and third authors have additional supervisory roles to ensure that this work is done perfectly. All authors made significant contributions to the manuscript. All authors read and approved the final manuscript.

## FUNDING INFORMATION

The study is funded as a PhD scholarship for the first author's. However, the funder had no role in study design, data collection, data analysis or manuscript writing and publication.

## CONFLICT OF INTEREST STATEMENT

The authors have no conflicts of interest to disclose.

## ETHICS STATEMENT

This study has obtain the ethical approval from Institutional Review Board of Muhimbili University of Health and Allied Sciences (ID: DA.282/298/01.C/).

## Data Availability

Data used in this study are accessible online.
